# The *Bacillus rugosus* BPH-S36 genome, a symbiont enabling brown planthopper *(Nilaparvata lugens*) adaptation to resistant rice

**DOI:** 10.1038/s41597-026-07541-4

**Published:** 2026-06-01

**Authors:** Wang Weixia, Zhang Jinli, Wei Qi, He Jiachun, Wan Pinjun, Zhu Tingheng

**Affiliations:** 1 Research and Development Center of Rice Cropping Technology, China National Rice Research Institute, Hangzhou, 311401 China; 2 College of Biotechnology and Bioengineering, Zhejiang University of Technology, Hangzhou, 310014 China

## Abstract

The brown planthopper (BPH, *Nilaparvata lugens*) is a major pest of rice, and its adaptation to resistant rice varieties often involves symbiotic microorganisms. *Bacillus rugosus* strain BPH-S36, isolated from a BPH population IR56 (virulent on resistant rice variety IR56), has been shown to enhance host survival on resistant rice when introduced into a susceptible BPH population TN1. To understand the genetic basis of this symbiotic virulence, we sequenced and assembled the complete genome of BPH-S36. The genome comprises a single circular chromosome of 4,120,256 bp with a GC content of 43.81%, encoding 4,288 protein-coding genes, 30 rRNA genes, 87 tRNA genes, and numerous genes related to carbohydrate metabolism, transport, and secondary metabolite synthesis. The assembly exhibits high completeness (98.4% BUSCO) and the genome sequence has been deposited in public databases (NCBI accession number JBSTRU000000000, BioProject: PRJNA1372835, BioSample: SAMN53627811). This high-quality genome resource provides a foundation for elucidating the molecular mechanisms underlying insect-symbiont-plant interactions and offers potential targets for novel pest management strategies.

## Background & Summary

Rice (*Oryza sativa* L.) is a staple food for more than half of the world’s population, and its stable production is crucial for global food security^1^. However, rice crops face persistent threats from various pests. Among them, the brown planthopper (BPH), *Nilaparvata lugens* (Stål), stands out as a devastating insect pest that has caused recurring outbreaks and significant yield losses across Asia^2,3^. A key feature of BPH is its ability to migrate over long distance^4^ and rapidly adapt to BPH-resistant rice varieties, frequently leading to the breakdown of host-plant resistance^5^. The molecular mechanisms underlying this virulence adaptation, a process known as biotype evolution, remain a central and unresolved question in plant-insect interactions^6^. Traditional research has largely focused on a direct arms race between rice and BPH, in this paradigm rice resistance genes (e.g., *Bph14*) mediate the recognition of BPH feeding and trigger defense responses, which the BPH then attempts to suppress via effector molecules in its saliva^7–11^.

Recent studies, however, have revealed that BPH harbors symbiotic microorganisms, primarily the yeast-like symbionts (YLS), *Arsenophonus* and *Wolbachia*. These symbionts are no longer viewed merely as nutritional partners, instead, they are increasingly recognized as active contributors to modulating the insect’s reproduction, virulence, detoxification processes, and overall fitness on resistant rice^12–18^, Notably, the heritable bacterial symbiont *Arsenophonus* has also been linked to insecticide resistance^19^.

Our previous work isolated a *Bacillus* strain (BPH-S36) from BPH population IR56(adapted to the resistant rice variety IR56). *In situ* hybridization showed the BPH-S36 were mainly distributed in the salivary glands, gut, fat body and female internal genitalia of BPH. Bioassays demonstrated that introducing this symbiont into a BPH population TN1 (specific to susceptible rice variety Taichung Native 1) significantly improved its survival rate from 52.1% ± 1.5% to 64.2% ± 3.0% and feeding ability on the resistant rice variety IR56. These results underscore the symbiont’s important role in BPH adaptation to resistant rice^20^. Due to the effects the symbiont has on BPH virulence, sequencing and analyzing the bacterial genome is paramount and has practical pest management applications.

## Method

### Sample collection and genome sequencing

**Fig. 1:**
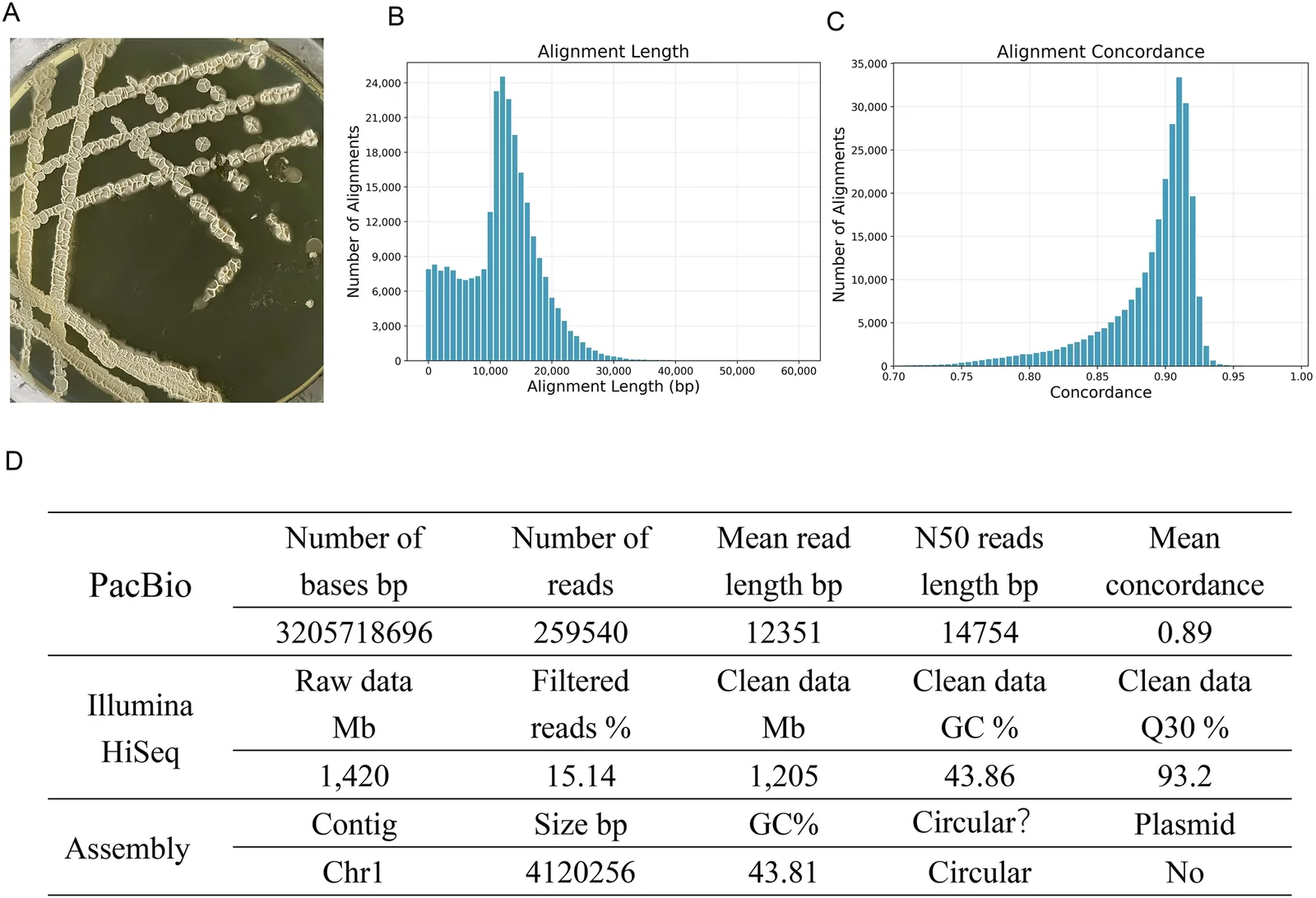
Morphology and sequencing data information of BPH-S36. (**A**) Morphology of the isolated BPH-S36 strain on an LB plate. (**B**) Read length distribution. (**C**) Aligment concordance. (**D**) PacBio and Illumina PE150 sequencing data statistics.

The IR56 and TN1 populations of BPH, originally collected from rice fields in China National Rice Research Institute (30.4°N, 119.5°E, Fuyang Zhejiang, 2010), have been reared for more than 130 generations in the laboratory on the resistant rice variety IR56 (*Bph3*) and the susceptible variety Taichung Native 1 (no resistant gene), respectively. Previously, a bacterial strain named BPH-S36 was isolated from the IR56 population on May 8, 2023 and functionally demonstrated to contribute to the population’s adaptation to the resistant rice variety IR56. The bacteria were identified as belonging to Bacillaceae. The morphological characteristics of the isolated bacterial strain BPH-S36 cultivated on an LB agar plate are presented in the Fig. 1A. This bacterial strain was preserved in 20% glycerol at -80°C. Genomic DNA was extracted with the STE method from an overnight culture of glycerol-recovered BPH-S36 in liquid LB medium (containing 0.5% yeast extract, 1.0% peptone, 1.0% NaCl) at 37 °C. Briefly, the bacterial pellet was resuspended in STE buffer (100 mM NaCl, 1 mM EDTA, 10 mM Tris-Cl, pH 8.0), followed by the addition of 1 mg/mL proteinase K and SDS to a final concentration of 0.5%. After incubation at 56 °C for 2 h with frequent mixing, RNase was added and incubated at 37 °C for 30 min. Genomic DNA was purified by two successive extractions with an equal volume of phenol: chloroform: isoamyl alcohol (25:24:1, v/v/v), and then extraction with equal volume of chloroform: isoamyl alcohol (24:1, v/v/v). Genomic DNA was then precipitated from the supernatant by adding 0.7 volumes of isopropanol. After washing the pellet with 70% ethanol, genomic DNA was recovered in TE buffer (1 mM EDTA, 10 mM Tris HCl pH 8.0). The harvested DNA was detected by the agarose gel electrophoresis and quantified by Qubit. High throughput DNA sequencing was performed on PacBio and Illumina PE150 by Beijing Novogene Bioinformatics Technology Co., Ltd.

### Genome assembly and evaluation

The genome was assembled from raw PacBio reads using Canu:(https://github.com/marbl/canu/,version:2.0). Draft CANU assembly was evaluated using QUAST 5.0.2^21^. Final assembly was polished with Illumina reads in a single round using Pilon v. 1.24^22^. A total of 3.206 Gbp of PacBio HiFi reads with a mean length of read 12351 bp and 1420 Mb Illumina reads with quality of Q30 93.2% were assembled to generate a single, circular chromosome of 4,120,256 bp. The read length distribution, alignment concordance, number of bases, reads and mean length of reads were shown in Fig. 1B–D. We evaluated the completeness of the genome assembly using Benchmarking Universal Single-Copy Orthologs (BUSCO) v2.0. The genome assembly was found to have a high level of completeness (98%).

### Gene prediction and functional annotation

**Fig. 2:**
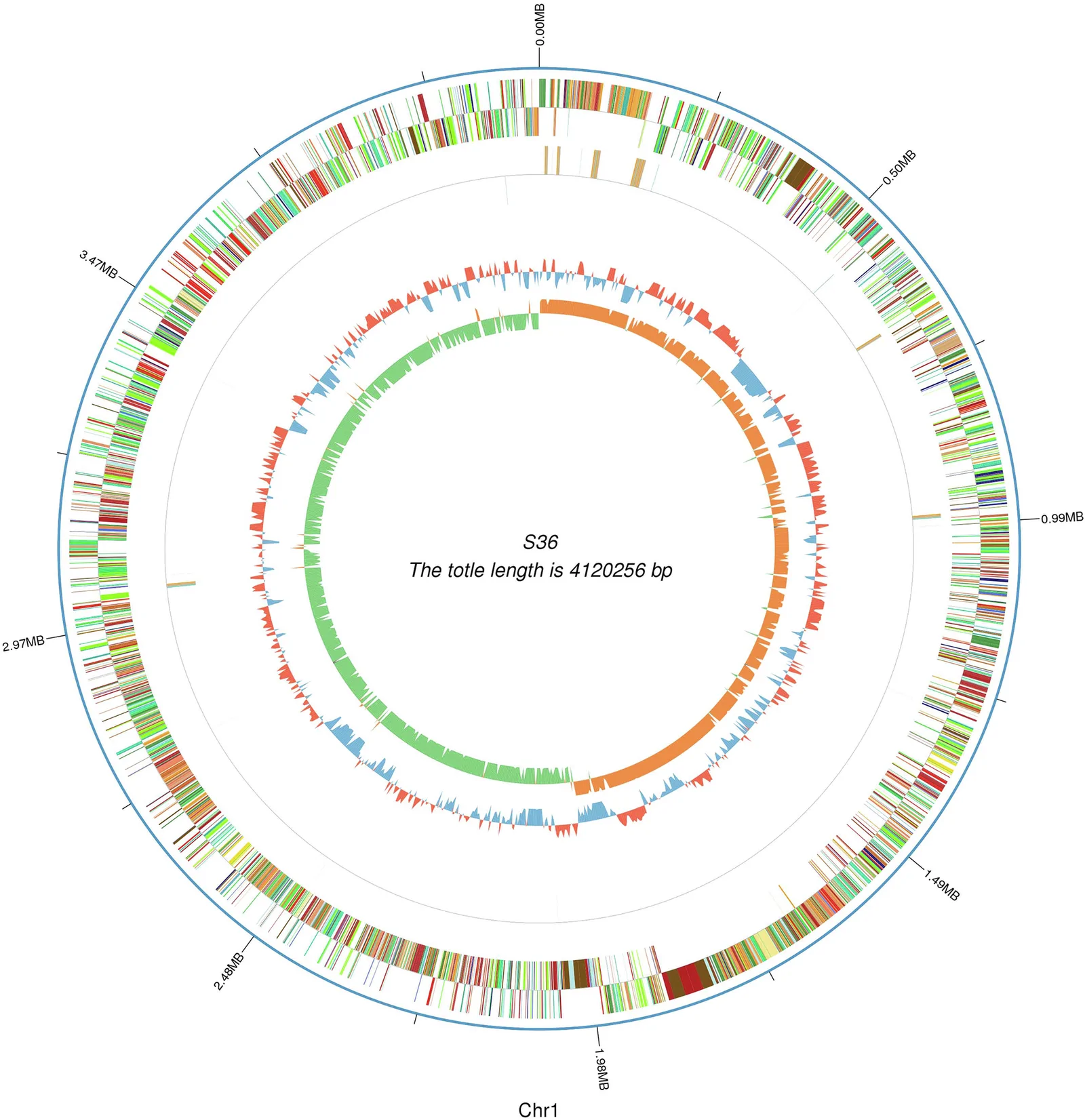
Circular plot of BPH-S36. The outermost circle displays genomic sequence position coordinates. Moving inward, the subsequent layers include coding genes, annotation information from the COG database and Non-coding RNA (ncRNA), with each represented by distinct colors corresponding to different annotation categories. Within the two innermost circles, the outer circle represents genomic GC content. The inward blue regions indicate GC content lower than the genome-wide average, while the outward red regions indicate the opposite. Higher peaks represent greater deviations from the average GC content. The inner circle represents the genomic GC skew value. The inward green regions indicate a lower G content relative to C, while the outward orange regions indicate the opposite.

Genome component prediction included the prediction of the coding gene with GeneMarkS program, repetitive sequences using the RepeatMasker (Version open-4.0.5) (http://www.repeatmasker.org/ and TRF (Tandem repeats finder Version 4.07b)^23^, non-coding RNA by the tRNAscan-SE (Version 1.3.1), rRNAmmer (Version 1.2) or BLAST against the Rfam database followed by using the cmsearch program (Version 1.1rc4) with default parameters to confirm the final set of sRNAs^24–26^, genomics islands with IslandPath-DIOMB program (Version 0.2)^27^, transposon with transposon PSI and clustered regularly interspaced short palindromic repeat sequences (CRISPR)^28^. Ultimately, we identified 4,288 protein-coding genes, which account for 89.1% of the genome sequence and 30 rRNA, 87 tRNA genes, and various repetitive sequences (Table 1). A circular genome map visualizes these features in Fig. 2.

**Table 1:** Summary of ncRNA and repetitive sequence in the genome of BPH-S36.

Type		Number	Avg Len	Total Len	% in Genome
ncRNA	tRNA	87	77	6,717	0.163
	5 s rRNA	10	115	1,150	0.0279
	16 s rRNA	10	1,539	15,388	0.3735
	23 s rRNA	10	2,928	29,283	0.7107
	sRNA	0	0	0	0
Repetitive sequence	LTR	174	74	12,890	0.3128
	LINE	63	60	3,792	0.092
	SINE	13	107	1,335	0.0324
	DNA transposon	44	65	2,765	0.0671
	RC	3	61	183	0.0044
	TR	43	6~282	4,476	0.1086
	Minisatellite DNA	34	10~60	2,387	0.0579
	Microsatellite DNA	1	6~6	30	0.0007

^1^LTR, Long Terminal Repeat. ^2^LINE, Long Interspersed Nuclear Elements. ^3^SINE, Short Interspersed Nuclear Elements. ^4^RC, rolling circle. ^5^TR, Tandem Repeats.

**Fig. 3:**
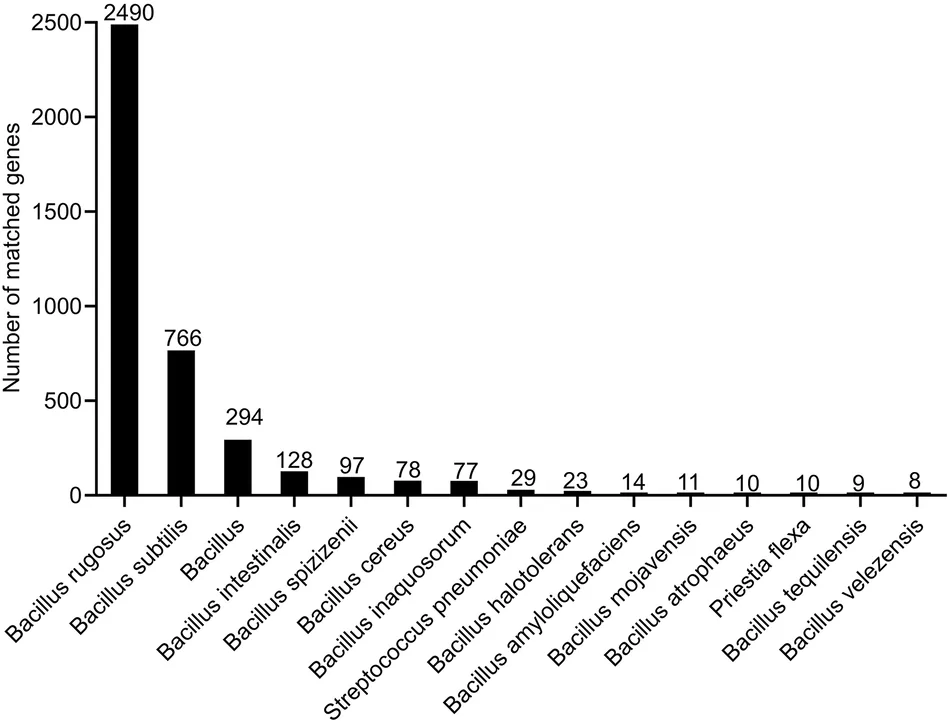
Statistical chart of species annotation for BPH-S36 based on the NR database. X-axis: Species name; Y-axis: Number of annotated genes.

Eight databases were used to predict gene functions. They were respective NR (Non-Redundant Protein Database databases), GO (Gene Ontology), KEGG (Kyoto Encyclopedia of Genes and Genomes), COG (Clusters of Orthologous Groups), Pfam (Protein families database), Swiss-Prot (http://www.expasy.ch/sprot), CAZy (Carbohydrate Active Enzymes database) and TCDB (Transporter classification database). A whole genome Blast search (E-value less than 1e-5, minimal alignment length percentage larger than 40%) was performed against above seven databases. The secretory proteins were predicted by the SignalP database^29^. Functional annotation revealed that 96.5% of the genes could be assigned putative functions across multiple databases (Table 2). Taxonomic distribution of the best NR hits showed most genes were assigned to *Bacillus rugosus* and *Bacillus subtilis* (Fig. 3).

**Table 2:** Functional annotation statistics of BPH-S36 genomes.

Sample name	Total gene No.	All Annotated Gene No.	Gene Annotated Coverage	Data base							
				NR	KEGG	Go	Pfam	SP	COG	CAZy	TCDB
BPH-S36	4288	4138	96.5%	4105	4025	2904	2904	3728	3229	185	593

**Fig. 4:**
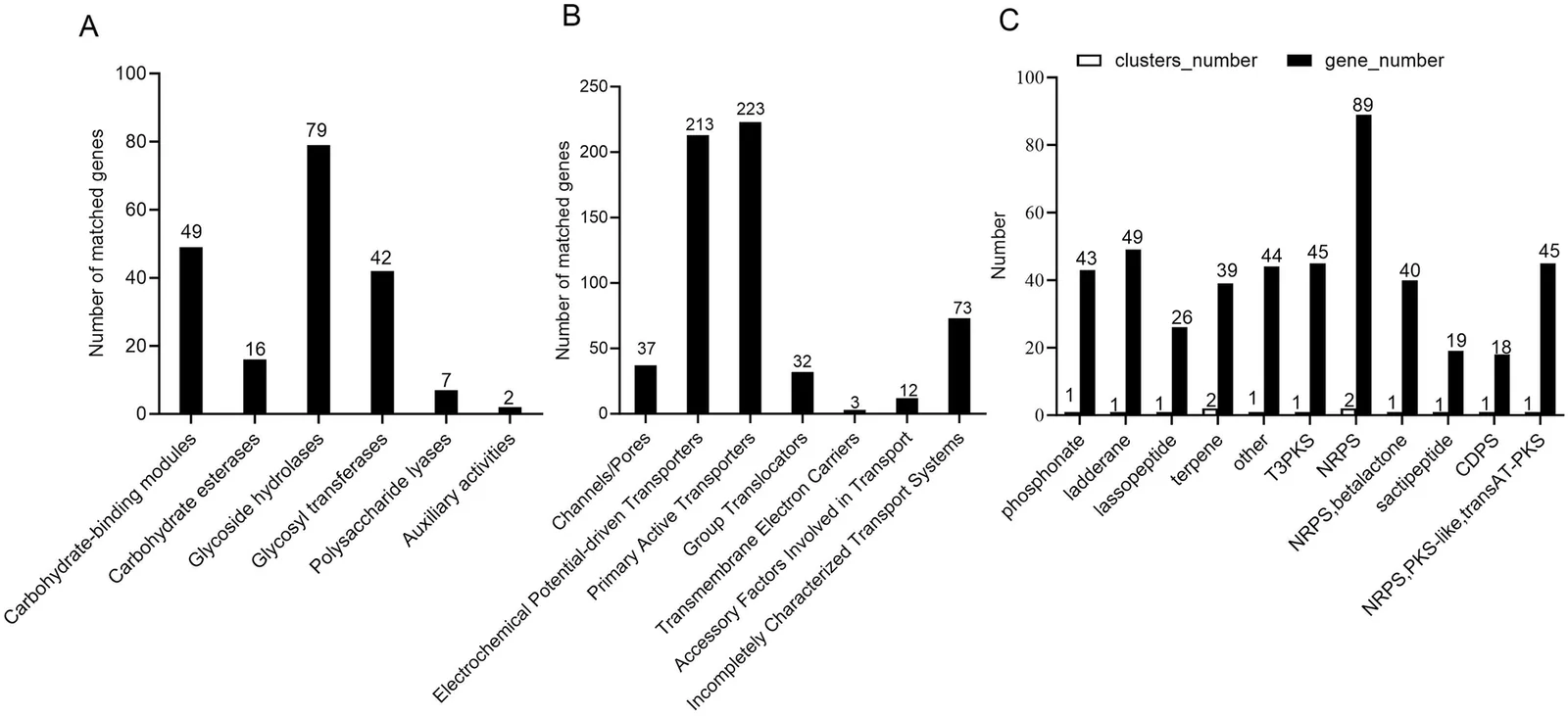
Distribution of secondary metabolite biosynthetic gene clusters and their sizes across BPH-S36. (**A**) Gene counts by CAZy category. X-axis, CAZy category. Y-axis, number of annotated genes. (**B**) Gene counts by TCDB category. (**C**) Secondary metabolic gene clusters and gene classification by antiSMASH-4.0.2.

Analysis using the CAZy and TCDB databases identified 185 genes involved in carbohydrate metabolism and 593 genes encoding transporter proteins (Fig. 4A,B). Notably, antiSMASH analysis predicted 13 gene clusters associated with secondary metabolite biosynthesis (Fig. 4C). Additionally, 149 putative secretory proteins, 1,148 transmembrane proteins and 99 proteins belong to Type III secretion system were predicted.

## Data Records

Assembled sequences along with gene annotation generated for this study are available from NCBI database under JBSTRU000000000^30^. The associated BioProject number is PRJNA1372835, and the BioSample number is SAMN53627811. Illumina raw reads can be directly accessed on NCBI SRA database under SRR36387075^31^. PacBio raw reads can be directly accessed on NCBI SRA database under SRR36447587^32^.

## Technical Validation

The quality of the BPH-S36 genome assembly was assessed by computing several metrics: (i) comparison with the estimated genome size, which is ~4.2 Mb (see above); (ii) genomic BUSCO analyses, which identified 98.4% of Bacillales BUSCOs in the BPH-S36 genome (98.2% are single-copied and 0.2% are duplicated, Table 3). The quality metrics indicated that theBPH-S36 genome assembly has a high level of completeness and is of high-quality.

**Table 3:** BUSCO assessment of the BPH-S36 genome assembly.

Total BUSCO groups searched	617	
Missing BUSCOs (M)	4	0.6%
Fragmented BUSCOs (F)	6	1.0%
Complete and duplicated BUSCOs (D)	1	0.2%
Complete and single-copy BUSCOs (S)	606	98.2%
Complete BUSCOs (C)	607	98.4%

## Data Availability

Assembled sequences along with gene annotation, have been deposited at NCBI database as JBSTRU000000000(2025). The associated BioProject number is PRJNA1372835, and the BioSample number is SAMN53627811. Illumina raw reads have been deposited to NCBI SRA database as SRR36387075(2025). PacBio raw reads have been deposited to NCBI SRA database as SRR36447587(2025).

## Code availability

All software and pipelines were executed according to the manual and protocols of the published bioinformatic tools. The version and code/parameters of software have been described in the Methods section.
